# Broad Spectrum Pro-Quorum-Sensing Molecules as Inhibitors of Virulence in Vibrios

**DOI:** 10.1371/journal.ppat.1002767

**Published:** 2012-06-28

**Authors:** Wai-Leung Ng, Lark Perez, Jianping Cong, Martin F. Semmelhack, Bonnie L. Bassler

**Affiliations:** 1 Department of Molecular Biology, Princeton University, Princeton, New Jersey, United States of America; 2 Howard Hughes Medical Institute, Chevy Chase, Maryland, United States of America; 3 Department of Chemistry, Princeton University, Princeton, New Jersey, United States of America; Harvard Medical School, United States of America

## Abstract

Quorum sensing (QS) is a bacterial cell-cell communication process that relies on the production and detection of extracellular signal molecules called autoinducers. QS allows bacteria to perform collective activities. *Vibrio cholerae*, a pathogen that causes an acute disease, uses QS to repress virulence factor production and biofilm formation. Thus, molecules that activate QS in *V. cholerae* have the potential to control pathogenicity in this globally important bacterium. Using a whole-cell high-throughput screen, we identified eleven molecules that activate *V. cholerae* QS: eight molecules are receptor agonists and three molecules are antagonists of LuxO, the central NtrC-type response regulator that controls the global *V. cholerae* QS cascade. The LuxO inhibitors act by an uncompetitive mechanism by binding to the pre-formed LuxO-ATP complex to inhibit ATP hydrolysis. Genetic analyses suggest that the inhibitors bind in close proximity to the Walker B motif. The inhibitors display broad-spectrum capability in activation of QS in *Vibrio* species that employ LuxO. To the best of our knowledge, these are the first molecules identified that inhibit the ATPase activity of a NtrC-type response regulator. Our discovery supports the idea that exploiting pro-QS molecules is a promising strategy for the development of novel anti-infectives.

## Author Summary

The disease cholera, caused by the pathogenic bacterium *Vibrio cholerae*, is a major health concern in developing regions. In order to be virulent, *V. cholerae* must precisely control the timing of production of virulence factors. To do this, *V. cholerae* uses a cell-cell communication process called quorum sensing to regulate pathogenicity. In the current work, we identify and characterize new classes of small molecules that interfere with quorum-sensing-control of virulence in multiple *Vibrio* species. The molecules target the key quorum-sensing regulator LuxO. These molecules have the potential to be developed into new anti-infectives to combat infectious diseases of global importance.

## Introduction

Quorum sensing (QS) is a process of bacterial cell-cell communication that relies on the production, release, detection, and response to extracellular signaling molecules called autoinducers. QS allows groups of bacteria to synchronously alter behavior in response to changes in the population density and species composition of the vicinal community. QS controls collective behaviors including bioluminescence, sporulation, virulence factor production, and biofilm formation (Reviewed in [1], [2]). Impairing virulence factor production or function has gained increasing attention as a method to control bacterial pathogenicity. The advantage of anti-virulence strategies over traditional antibiotics is presumed to be reduced pressure on bacteria to develop resistance [3]–[5]. Because QS controls virulence in many clinically relevant pathogens, disrupting QS is viewed as a promising possibility for this type of novel therapeutic development [6]–[8].

Many pathogenic Gram-negative bacteria use acylhomoserine lactones (HSLs) as QS autoinducers, which are detected by either cytoplasmic LuxR-type or membrane-bound LuxN-type receptors [9]. To date, efforts to interfere with HSL QS in Gram-negative bacteria have yielded several potent antagonists [10]–[15]. While these strategies are exciting, some globally important Gram-negative pathogens do not use HSLs as autoinducers. Thus, additional strategies that target non-HSL based QS systems are required. Here, we describe the identification and characterization of a set of small-molecule inhibitors that act on the non-HSL QS system of *Vibrio cholerae* by targeting two independent steps in the signal transduction pathway.

**Figure 1:**
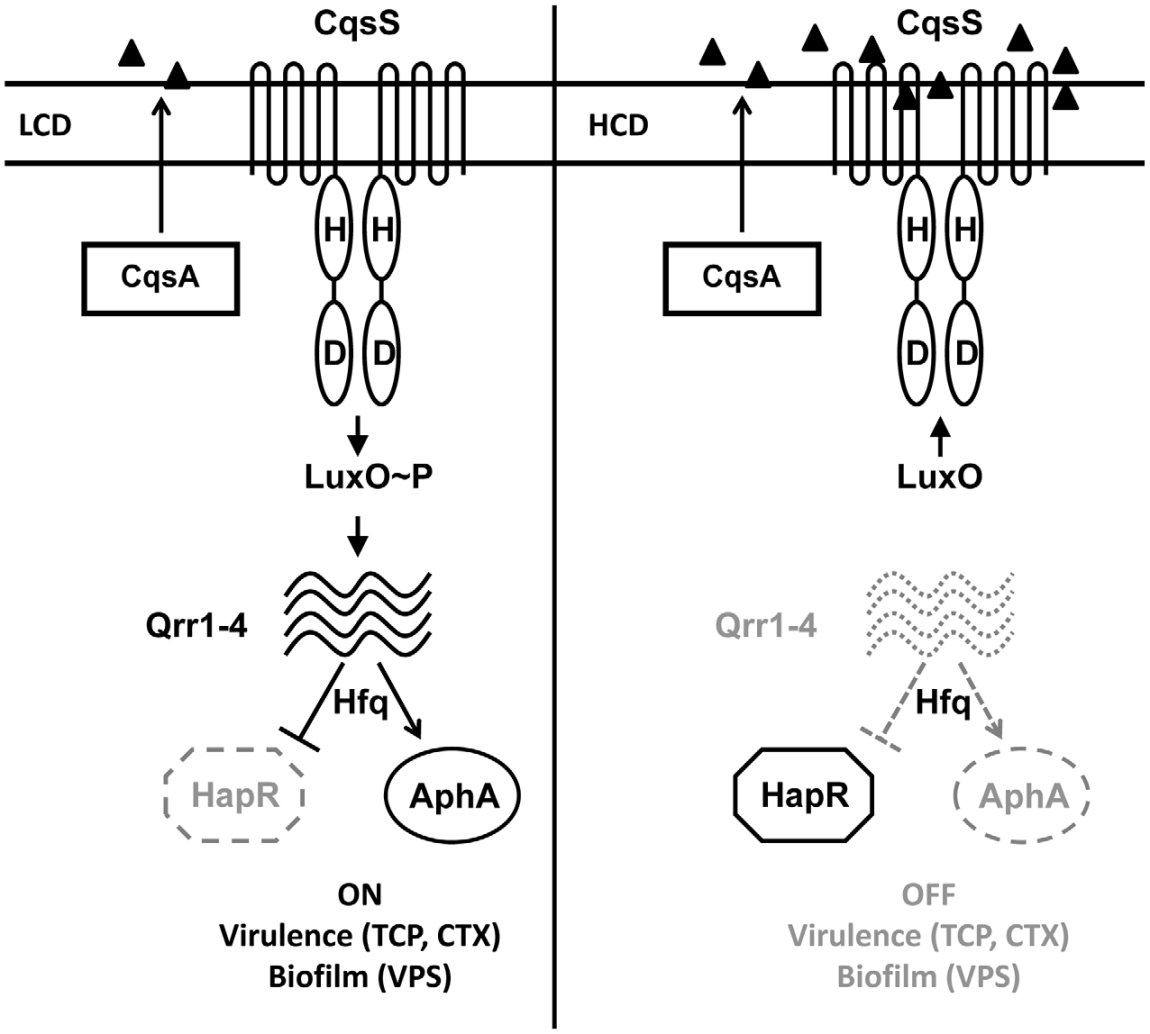
The Quorum-Sensing Circuit in *Vibrio cholerae*. The CqsA/CqsS signal transduction system is shown as the example for the *V. cholerae* QS circuit. (Left) At low cell density (LCD), the CAI-1 autoinducer concentration is below the detection threshold, and the membrane bound CqsS receptor functions as a kinase. The LuxO response regulator is phosphorylated and it activates the transcription of genes encoding the four Qrr sRNA genes. Aided by the RNA chaperone Hfq, the Qrr sRNAs activate and repress translation of the AphA and HapR proteins, respectively. (Right) At high cell density (HCD), binding of CAI-1 to CqsS inhibits its kinase activity. LuxO is not phosphorylated and transcription of the *qrr* genes is terminated. Translation of AphA is inhibited and HapR is derepressed. Hundreds of genes are controlled by AphA and HapR, including genes required for biofilm formation and virulence. HapR also functions as a transcriptional activator of the heterologous *V. harveyi lux* operon [22], [24], [26]–[30]. Dotted lines denote components that are not expressed while solid lines represent those that are produced.

*V. cholerae* is the etiological agent of the disease cholera and its annual global burden is estimated to be several million cases [16]. *V. cholerae* produces and detects two QS autoinducer molecules called CAI-1 and AI-2. CAI-1 ((*S*)-3-hydroxytridecan-4-one) is produced by the CqsA synthase [17], [18] and AI-2 ((2*S*, 4*S*)-2-methyl-2,3,3,4-tetrahydroxytetrahydrofuran borate) is produced by the LuxS synthase [19], [20]. Detection of CAI-1 and AI-2 occurs through transmembrane receptors CqsS and LuxPQ, respectively [21], [22]. CqsS and LuxPQ are two-component proteins that possess both kinase and phosphatase activities (Figure 1 shows the CqsA/CqsS system). At low cell density (LCD), when the receptors are devoid of their respective ligands, their kinase activities predominate, resulting in the phosphorylation of the response regulator LuxO. LuxO∼P is the transcriptional activator of four genes encoding small regulatory RNAs (sRNAs), Qrr1-4 [23]. The Qrr sRNAs target the mRNAs encoding the quorum-sensing master transcriptional regulators AphA and HapR. At LCD, facilitated by the RNA chaperone Hfq, Qrr1-4 stabilize and destabilize the *aphA* and *hapR* mRNA transcripts, respectively [23]. Therefore, AphA protein is made while HapR protein is not (Figure 1). When autoinducer concentration increases above the threshold required for detection (which occurs at high cell density (HCD)), binding of the autoinducers to their cognate receptors switches the receptors from kinases to phosphatases (Figure 1). Phosphate flow through the signal transduction pathway is reversed, resulting in dephosphorylation and inactivation of LuxO. Therefore, at HCD, *qrr*1-4 are not transcribed, resulting in cessation of translation of *aphA* and derepression of translation of *hapR*. This QS circuitry ensures maximal AphA production at LCD and maximal HapR production at HCD. AphA and HapR each control the transcription of hundreds of downstream target genes [24], [25]. Hence, reciprocal gradients of AphA and HapR establish the QS LCD and HCD gene expression programs, respectively (Figure 1).

In pathogens that cause persistent infections, QS commonly activates virulence factor production at HCD. However, in *V. cholerae*, which causes an acute disease, HapR production at HCD *represses* genes important for biofilm formation and virulence factor production [22], [26]–[30]. This peculiar pattern of virulence gene regulation can be understood in terms of the disease caused by *V. cholerae* [31]. Following successful *V. cholerae* infection, the ensuing diarrhea washes huge numbers of bacteria from the human intestine into the environment. Thus, expression of genes for virulence and biofilm formation at LCD promotes infection, while repression of these genes by autoinducers at HCD promotes dissemination. Thus, molecules that *activate* QS have the potential to repress virulence in *V. cholerae*. Moreover, QS plays an essential role in virulence in other pathogenic vibrios including *Vibrio parahaemolyticus*, *Vibrio alginolyticus*, and *Vibrio vulnificus* [32]–[35]. The components of the QS circuits in these species are similar to those of *V. cholerae*. Therefore, QS-activating molecules identified for *V. cholerae* could be broadly useful for controlling diseases caused by other vibrios.

Here, we report the identification of a set of small molecules that activate the QS system of *V. cholerae*. We classify the QS-activating molecules as either QS receptor agonists or LuxO inhibitors. Because we have already reported analyses of QS receptor agonists, we focus here on the LuxO inhibitors. At LCD, LuxO∼P activates production of the Qrr sRNAs, which repress HapR; inhibitors of LuxO thus *activate* QS due to derepression of HapR. LuxO belongs to the NtrC protein family, σ^54^-binding transcriptional activators that rely on ATP hydrolysis to promote open complex formation [36]. The LuxO inhibitors identified here function uncompetitively to perturb LuxO ATPase activity. Genetic analysis of LuxO mutants that are insensitive to the inhibitors suggests that the inhibitors interact with a region adjacent to the ATP binding pocket. Finally, using a set of phenotypic assays, we show that the inhibitors broadly activate different vibrio QS circuits and, in turn, repress virulence factor production and reduce cytotoxicity. Because LuxO is conserved among vibrio QS circuits, the molecules we characterize here are capable of inhibiting HSL-based and non-HSL-based vibrio QS systems. Numerous NtrC-type proteins homologous to LuxO act in two-component signaling systems and their roles in controlling nitrogen metabolism, virulence, motility, and other important processes have been extensively studied (Reviewed in [37]). To the best of our knowledge, there exists no previous report of a chemical probe that modulates the activity of a NtrC-family response regulator.

## Results

### Identification of molecules that activate QS in *V. cholerae*

**Figure 2:**
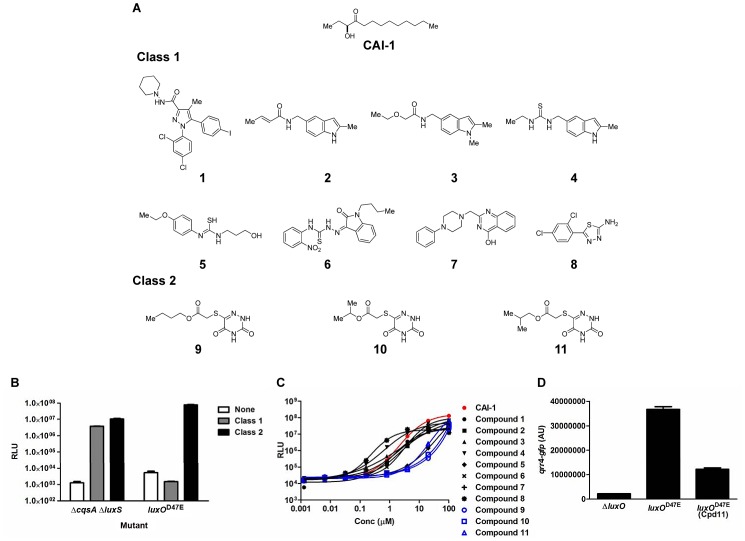
Identification of QS-activating compounds in *V. cholerae*. (A) Chemical structures of the eleven QS-activating compounds. The structure of CAI-1 is shown for reference. (B) Differential responses to Class 1 and Class 2 compounds by the *V. cholerae* Δ*cqsA* Δ*luxS* double synthase mutant (BH1578) and the *luxO* ^D47E^ mutant (BH1651). The normalized light (RLU, relative light units) produced was monitored in the absence (white) and presence of Class 1 (gray) or Class 2 (black) compounds. A representative experiment is shown using compound 1 (Class 1) and compound 11 (Class 2) from (A). (C) QS dose-response curves of *V. cholerae*. The normalized light (RLU, relative light units) produced by the *V. cholerae* Δ*cqsA* Δ*luxS* mutant carrying the *lux* operon (BH1578) is plotted as a function of the concentration of the eleven QS-activating compounds shown in (A). Black curves denote responses to Class 1 compounds. Blue curves denote responses to Class 2 compounds. The red curve denotes the response to the native autoinducer CAI-1, which is the positive control. Error bars are present, but are too small to be observed in the plot. The bars represent standard errors of the mean for three independent trials. (D) Effect of compound 11 on expression of *qrr*4. Expression of *qrr*4 was monitored in a *V. cholerae luxO* ^D47E^ strain carrying a *qrr*4-*gfp* transcriptional reporter (SLS353). The response is shown in the presence and absence of 50 µM compound 11. Expression of *qrr*4-*gfp* from the Δ*luxO* mutant (SLS373) is shown for reference. AU denotes arbitrary units. Error bars represent standard errors of the mean for three independent trials.

We are interested in identifying small molecules that activate QS in *V. cholerae*, in order to induce the HCD state and thus repress virulence factor production. To do this, we developed a whole-cell high-throughput screen that relies on QS-dependent induction of bioluminescence (*lux*) in *V. cholerae* [22]. We exploited *V. cholerae* mutants genetically locked into the LCD state and carrying the *lux* operon from *V. harveyi* to screen for molecules that induce light production, indicating that they activate QS responses. We performed the screen on two different LCD mutants. The first mutant lacks the two autoinducer synthases, CqsA and LuxS. Therefore, both CqsS and LuxPQ QS receptors function as kinases and constitutively phosphorylate LuxO, resulting in transcription of the Qrr regulatory RNAs, and repression of translation of HapR (see INTRODUCTION). In the absence of HapR, there is no transcription of the heterologous *lux* operon, and thus, this strain is dark. The second strain carries the *luxO* ^D47E^ allele. This *luxO* mutation mimics LuxO∼P, rendering LuxO constitutively active [23], [38]. Therefore, HapR is repressed and the strain is dark. We anticipated identifying two classes of molecules that could induce light production: Class 1) Molecules that induce bioluminescence in the double synthase mutant but not in the *luxO* ^D47E^ mutant. These compounds are predicted to be QS receptor agonists; and Class 2) Molecules that induce bioluminescence in both the double synthase mutant and the *luxO* ^D47E^ mutant. Class 2 compounds likely target QS components that lie downstream of the receptors. We screened 90,000 molecules and identified eight Class 1 compounds and three Class 2 compounds (Figures 2A and 2B). The EC_50_ of Class 1 compounds are comparable to that of CAI-1 and generally lower than those of Class 2 compounds (Figure 2C). These differences support the idea that the two classes of molecules potentiate QS responses by distinct mechanisms. None of the compounds affected cell growth (Figure S1).

### Investigation of the targets of the QS activating compounds

To determine which QS component each compound acts on, we first tested the eight Class 1 compounds against *V. cholerae* mutants that lack only the CqsS receptor or only the LuxPQ receptor. All eight Class 1 compounds induced light production in the Δ*luxPQ* strain but not the Δ*cqsS* strain; hence, these eight molecules function as CqsS agonists (Figure S2). Interestingly, none has structural homology to the native CAI-1 autoinducer [17], [18], [39], [40] (Figure 2A). The Class 1 molecules are currently being characterized and are not discussed further here.

The three Class 2 compounds that activate QS in both of the LCD screening strains likely act downstream of the QS receptors. These three compounds are structurally homologous (Figure 2A); therefore, they may function by an identical mechanism. Here, we focused on the compound displaying the highest potency (i.e., compound 11, Figures 2A and 2C). Class 2 compounds could potentially target one or more of the *V. cholerae* QS cytoplasmic components that function downstream of the receptors: LuxO, σ^54^, Hfq, and/or Qrr1-4. We reasoned that if these compounds interfere with LuxO or σ^54^, transcription of *qrr*1-4 would decrease in the presence of the inhibitors. By contrast, if the compounds target Hfq or act directly on Qrr1-4, they should not affect *qrr*1-4 transcription. GFP production from a *qrr*4-*gfp* transcriptional fusion decreased ∼3-fold when the *luxO* ^D47E^ strain was treated with compound 11 (Figure 2D). This result suggests that compound 11 targets either LuxO or σ^54^. If the target of compound 11 is σ^54^, transcription of other σ^54^-dependent genes should be affected when *V. cholerae* is treated with the compound. We examined transcription of the σ^54^-dependent gene *vpsR* [41] and found that it did not change significantly in the presence of compound 11 (data not shown). These results suggest that compound 11 targets LuxO.

### Structure-activity-relationship of Class 2 compounds

**Figure 3:**
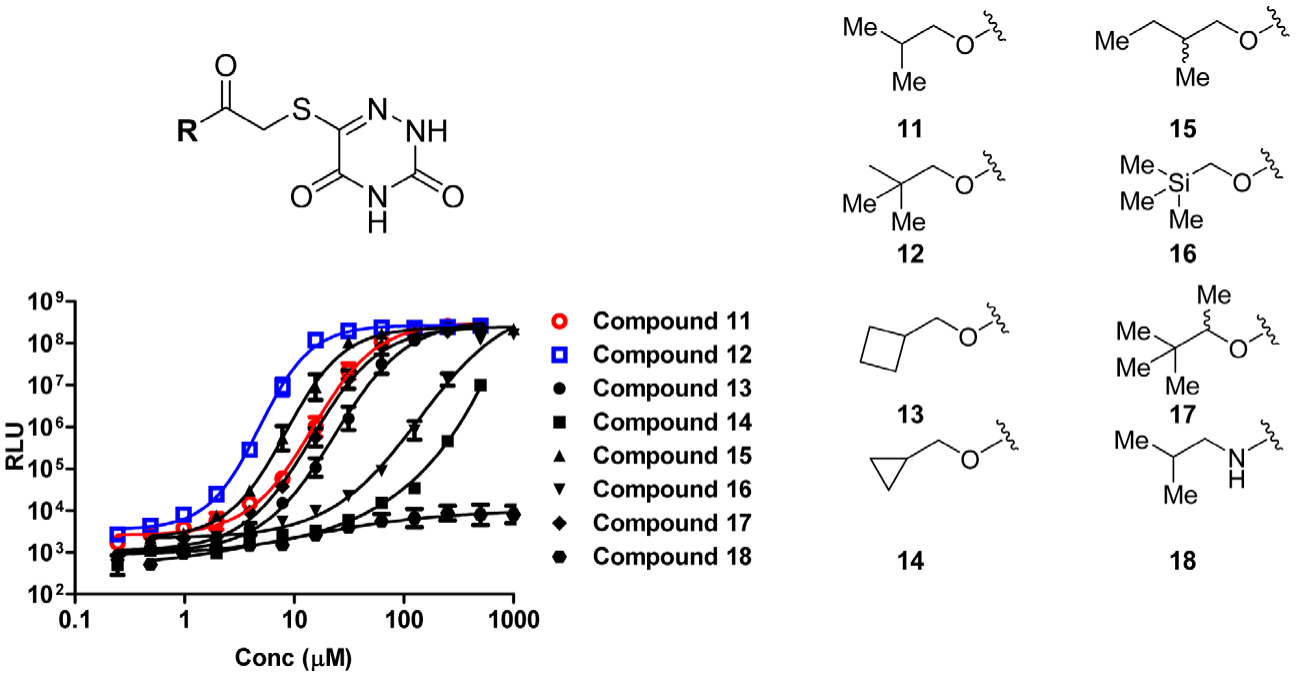
Structure-Activity-Relationship of LuxO inhibitors. The core chemical structure of the LuxO inhibitors is shown at the top. All analogs possess the identical 6-thio-5-azauracil moiety with modifications in the terminal side chains (denoted R). Variations in the side chain are shown on the right. Normalized light (RLU, relative light units) produced by the *V. cholerae luxO* ^D47E^ strain (BH1651) carrying the *lux* operon is plotted as a function of concentration of the eight different analogs. Error bars are present, but are too small to be observed in the plot. The bars represent standard errors of the mean for three independent trials.

The three identified Class 2 compounds share a 5-thio-6-azauracil core and only their side chains vary (Figure 2A). In addition, several 5-thio-6-azauracil analogs with other modifications on their side chains displayed weak or no activity in the screen. Therefore, differences in the hydrocarbon side chains must be responsible for the corresponding differences in potency with compounds harboring branched side chains displaying greater potency (i.e., compound 11, Figure 2C). To explore the relationship between structure and activity, we synthesized a focused library of compounds bearing the conserved 5-thio-6-azauracil core, and we altered the branching in the side chains. We measured activities using bioluminescence in the *V. cholerae luxO* ^D47E^ mutant. Several of the side chain modifications decreased potency (as shown by an increase in EC_50_, Figure 3). However, increasing steric bulk by incorporation of a *tert*-butyl carbinol side chain led to a 3-fold enhancement in potency (i.e., compound 12, Figure 3). Thus, the activity of the 5-thio-6-azauracil compounds within this series is highly sensitive to the structural features of the alkyl side chain. In the focused group of molecules we investigated, a bulky, hydrophobic terminal *t*-butyl moiety is optimal.

### Class 2 compounds inhibit the LuxO ATPase activity

NtrC-type response regulators including LuxO possess three biochemical activities: phosphoryl-group accepting activity, DNA-binding activity, and ATP hydrolyzing activity [36]. We investigated which of these activities is inhibited by compounds 11 and 12. First, using whole-cell bioluminescence assays, we found that both compounds activate QS in *V. cholerae* strains expressing either wild type LuxO or LuxO D47E (Figures 2B and 3). Wild type LuxO is activated by phosphorylation via the QS cascade, and the LuxO D47E variant, which mimics LuxO∼P, while not phosphorylated is constitutively active [22], [23], [26], [38]. Because both wild type LuxO and LuxO D47E are vulnerable to inhibition, it cannot be the ability of LuxO to participate in phosphorylation or dephosphorylation that is impaired by compounds 11 and 12.

**Figure 4:**
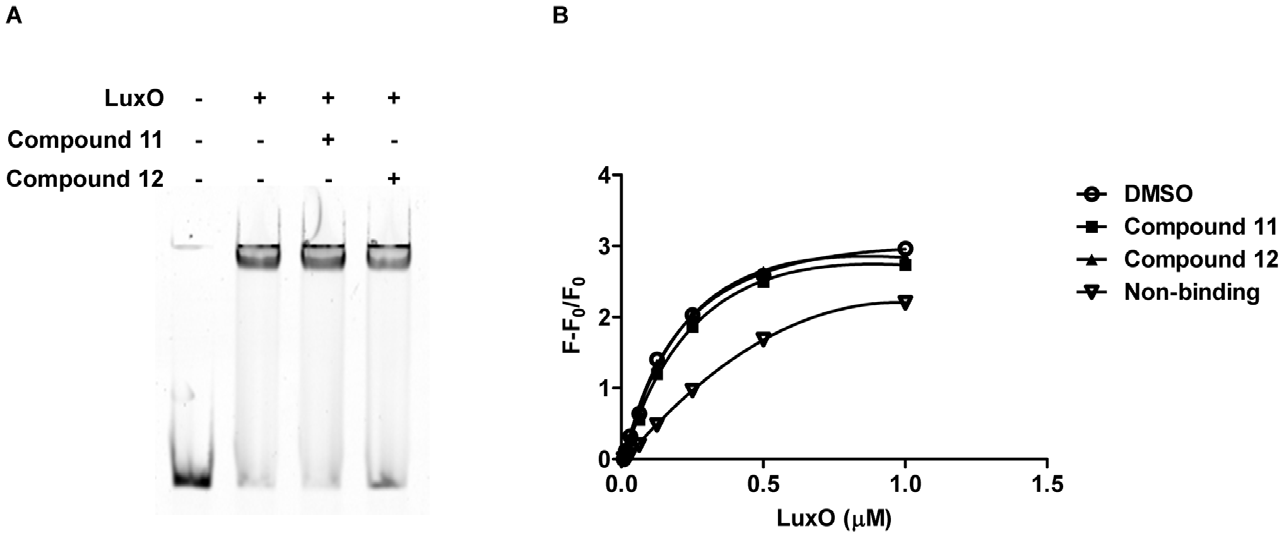
The LuxO Inhibitor does not affect DNA binding. LuxO D47E DNA binding in the presence and absence of compounds 11 and 12 was investigated by gel mobility shift assays (A) and fluorescent anisotropy assays (B). In (A), LuxO D47E was present at 1 µM. Compounds 11 and 12 were present at 200 µM. In (B), LuxO D47E was present at the indicated concentrations and compounds 11 and 12 were present at 200 µM. Error bars are present, but are too small to be observed in the plot. The bars represent standard errors of the mean for three independent trials.

LuxO, as a NtrC-type response regulator, binds to σ^54^-dependent promoters to activate transcription. Compounds 11 and 12 could prevent LuxO from binding to DNA, and in so doing, prevent *qrr* transcription. To investigate this possibility, we used electrophoretic-mobility-shift and fluorescence anisotropy assays to probe the LuxO interaction with *qrr* promoter DNA. Even in the presence of a high concentration (200 µM) of the inhibitors, no significant change in LuxO D47E binding to *qrr*4 promoter DNA occurred as judged by mobility shift (Figure 4A). Quantitative fluorescence anisotropy assays revealed that, in the presence and absence of the LuxO inhibitors, LuxO D47E interacts with the *qrr*4 promoter DNA with an identical binding constant (∼300 nM) (Figure 4B). Thus, binding to DNA is not altered by the inhibitors.

**Figure 5:**
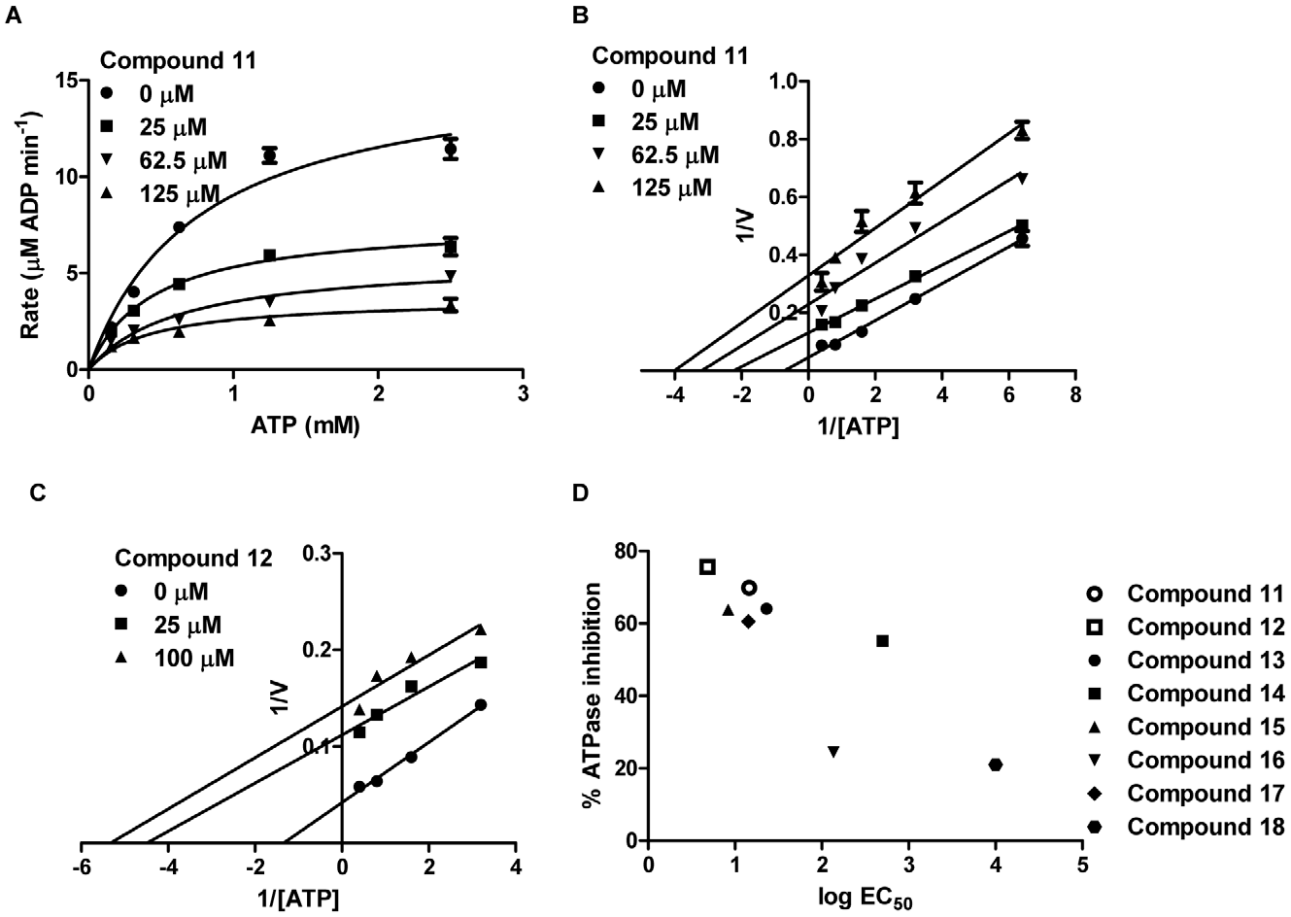
Enzyme kinetic analyses of LuxO ATPase inhibition. (A) Michaelis-Menton enzyme kinetic analysis of LuxO ATPase activity. The LuxO D47E ATP hydrolysis rate is plotted as a function of the concentration of ATP in the presence of the indicated amounts of compound 11. Error bars represent standard errors of the mean for at least three independent trials. (B) Lineweaver-Burk plot derived from the assay described in (A). (C) Lineweaver-Burk plot derived from a LuxO D47E ATPase assay in the presence of the indicated amounts of compound 12. (D) Correlation between % inhibition of LuxO D47E ATPase activity (2.5 mM ATP and 30 µM inhibitors) and EC_50_ of QS-activation potency (derived from Figure 3) for the different LuxO inhibitors.

Finally, we examined whether compounds 11 and 12 affect LuxO ATPase activity. To do this, we used a coupled-enzyme assay [42] to assess the rate of ATP hydrolysis by LuxO in the presence and absence of the compounds. Both compounds inhibit ATP hydrolysis in a dose-dependent manner (Figures 5A–C). Using traditional Michaelis-Menton enzyme kinetic analyses, we found that both compounds decrease the *K*_m_ and the *V*_max_ of the LuxO ATPase reaction (Figures 5B and 5C). The Lineweaver-Burk plots of curves derived from control reactions and from inhibitor-containing reactions display parallel slopes (*K*_m_/*V*_max_), indicating that compounds 11 and 12 function as uncompetitive inhibitors (Figures 5B and 5C), suggesting they bind to the pre-formed LuxO-ATP complex to inhibit ATP hydrolysis. Indeed, inhibition of LuxO ATPase by the analogs we identified or synthesized (as represented by % inhibition) is correlated with their potency (EC_50_) in inducing QS in the *lux*O^D47E^ mutant (Figure 5D). We conclude that the LuxO inhibitors discovered here activate QS in *V. cholerae* by specifically inhibiting the ATPase activity of LuxO. Presumably, in the presence of the inhibitors, LuxO is incapable of participating in open complex formation at the *qrr* promoters, which prevents transcription of the Qrr sRNAs. In turn, translation of HapR is derepressed and the QS response occurs prematurely.

### A genetic screen to identify LuxO mutants resistant to compound 12

**Figure 6:**
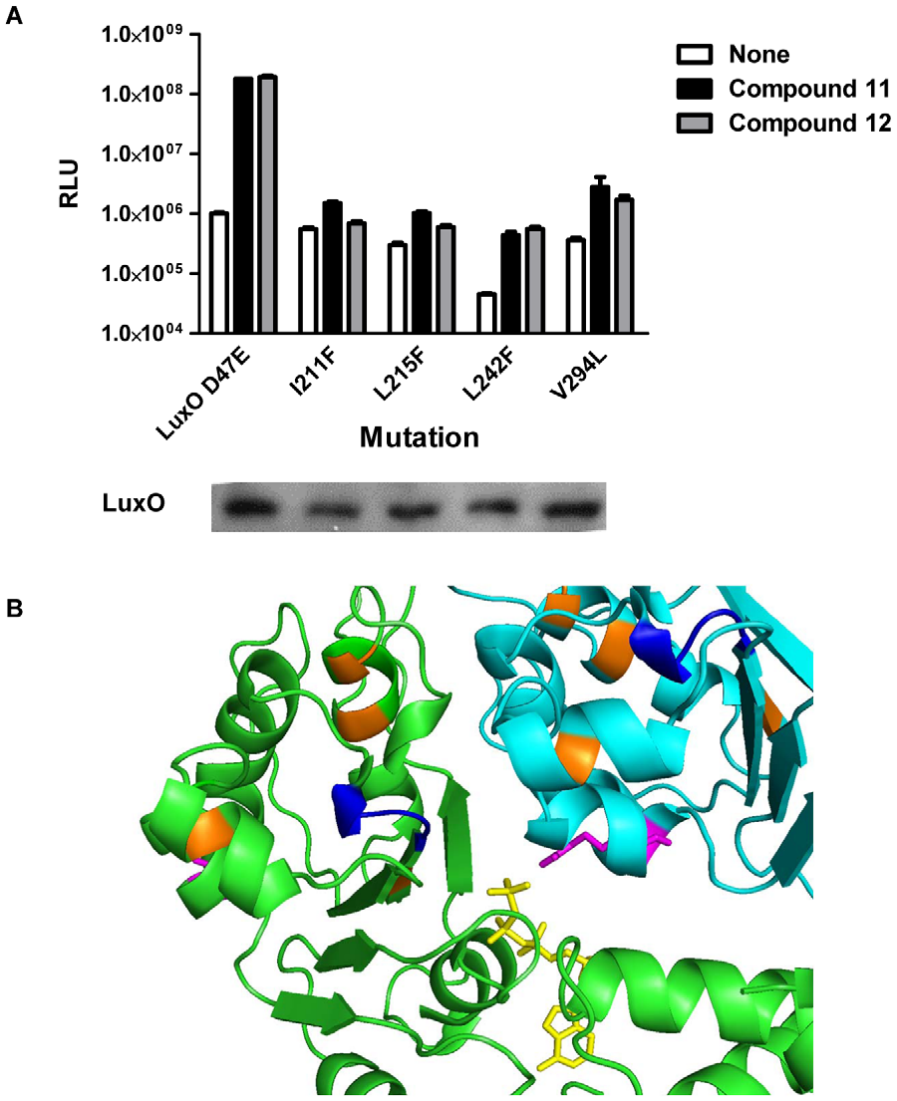
Isolation of LuxO mutants resistant to inhibition. (A) Normalized light (RLU, relative light units) produced by the *V. cholerae* Δ*luxO* strain carrying *luxO* ^D47E^ and *luxO* ^D47E^ harboring additional mutations in the absence (white) or presence of 100 µM of compound 11 (black) or compound 12 (gray). Error bars represent standard errors of the mean for three independent trials. Western blot analyses demonstrate that the wild type and all mutants produce comparable amounts of LuxO protein. (B) The locations of the resistance-conferring mutations are inferred from the ATP-bound *Aquifex aeolicus* NtrC1 structure (3M0E). Two monomers of NtrC1 are shown (cyan and green). The residues predicted to form the Walker B motif are shown in blue. The four resistance-conferring mutations (I211, L215, L242, and V294) are shown in orange. The catalytic arginine residue and ATP are shown in magenta (with side chain) and yellow, respectively.

Compounds 11 and 12 likely bind to LuxO at an allosteric site that negatively regulates ATP hydrolysis activity. To determine where compounds 11 and 12 bind, we screened for LuxO mutants refractory to inhibition. To do this, we engineered random mutations into the cloned *luxO* ^D47E^ gene and introduced the mutant library into a *V. cholerae* Δ*luxO* strain carrying the *lux* operon. We screened for clones that conferred a dark phenotype in the presence of compound 12, hypothesizing that such mutants harbor alterations in the inhibitor binding-site. Four such mutants were identified (Figure 6A). These LuxO D47E variants all possess an active ATPase and are functional, as judged by their ability to repress light production in the absence of inhibitor (Figure 6A). Sequencing revealed that the four LuxO D47E mutants carry I211F, L215F, L242F, or V294L alterations, implicating these residues as important for binding of the inhibitors. We mapped these four alterations onto the existing crystal structure of ATP-bound *Aquifex aeolicus* NtrC1 (PDB:3M0E) [43], which has high sequence homology to LuxO (Figure 6B). The four residues we identified in the screen map to three regions that abut the Walker B motif (D245, E246, L247, and C248 in LuxO) (Figure 6B). In other NtrC-type proteins, mutations in this region have been shown to prevent ATP hydrolysis (See DISCUSSION). These four *luxO* mutations were introduced into wild type LuxO and the resulting mutants are similarly resistant to inhibition (Figure S3). Thus, binding of compounds 11 and 12 to this region may induce a conformational change in the nearby ATP-binding pocket that inhibits ATP hydrolysis.

### Broad spectrum activation of vibrio QS

**Figure 7:**
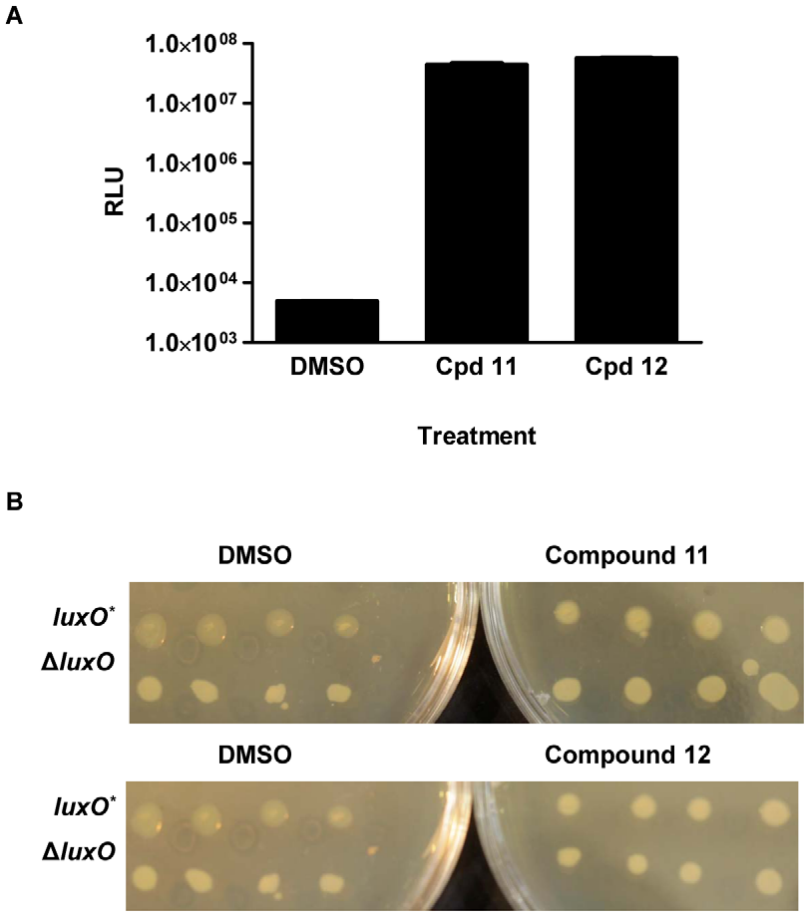
The LuxO inhibitors activate QS in different Vibiro species. (A) Normalized light (RLU, relative light units) produced by the *V. harveyi luxO* ^D47E^ strain in the absence and presence of 50 µM of compounds 11 and 12. (B) Colony morphology of the constitutively active V. parahaemolyticus luxO^*^ mutant (LM4476) and the isogenic *V. parahaemolyticus* Δ*luxO* mutant (LM9688) in the absence and presence of 500 µM compounds 11 and 12. Each strain was inoculated four times on the same plate.

As mentioned, LuxO is a conserved member of vibrio QS circuits. We therefore wondered if, similar to what we found in *V. cholerae*, compounds 11 and 12 could activate QS in other *Vibrio* species. To test this idea, we exploited two well-characterized phenotypes controlled by QS: light production in *V. harveyi* and colony opacity in *Vibrio parahaemolyticus* [44]–[46]. In *V. harveyi*, light production is induced by QS and a *V. harveyi luxO* ^D47E^ mutant is dark. Treatment of *V. harveyi luxO* ^D47E^ with compounds 11 and 12 induced light production 10,000-fold, indicating that these compounds are indeed active in *V. harveyi* (Figure 7A). In *V. parahaemolyticus*, the HapR ortholog, OpaR, controls colony opacity. OpaR production is repressed at LCD by LuxO∼P via the *V. parahaemolyticus* Qrr sRNAs. *V. parahaemolyticus* mutants that produce low and high levels of OpaR form translucent and opaque colonies, respectively [32], [46]. Thus, *V. parahaemolyticus* is naturally translucent at LCD and opaque at HCD. McCarter *et al* [32] recently identified a constitutively active LuxO mutant (LM4476, *luxO* ^*^) in *V. parahaemolyticus* that confers a constitutive translucent colony morphology (Figure 7B, left). By contrast, an isogenic *V. parahaemolyticus* Δ*luxO* strain (LM9688) forms opaque colonies (Figure 7B, left). When the *luxO* ^*^ mutant is plated on medium containing compound 11 or compound 12, the colonies switch from translucent to opaque, a phenotype indistinguishable from the Δ*luxO* mutant (Figure 7B, right). These results suggest that compounds 11 and 12 inhibit *V. parahaemolyticus* LuxO from repressing the OpaR-dependent QS program. We conclude that the LuxO inhibitors identified in this study are broadly capable of activating QS in *Vibrio* species that employ LuxO as the central QS regulator.

### New chemical tools for controlling virulence in vibrios

**Figure 8:**
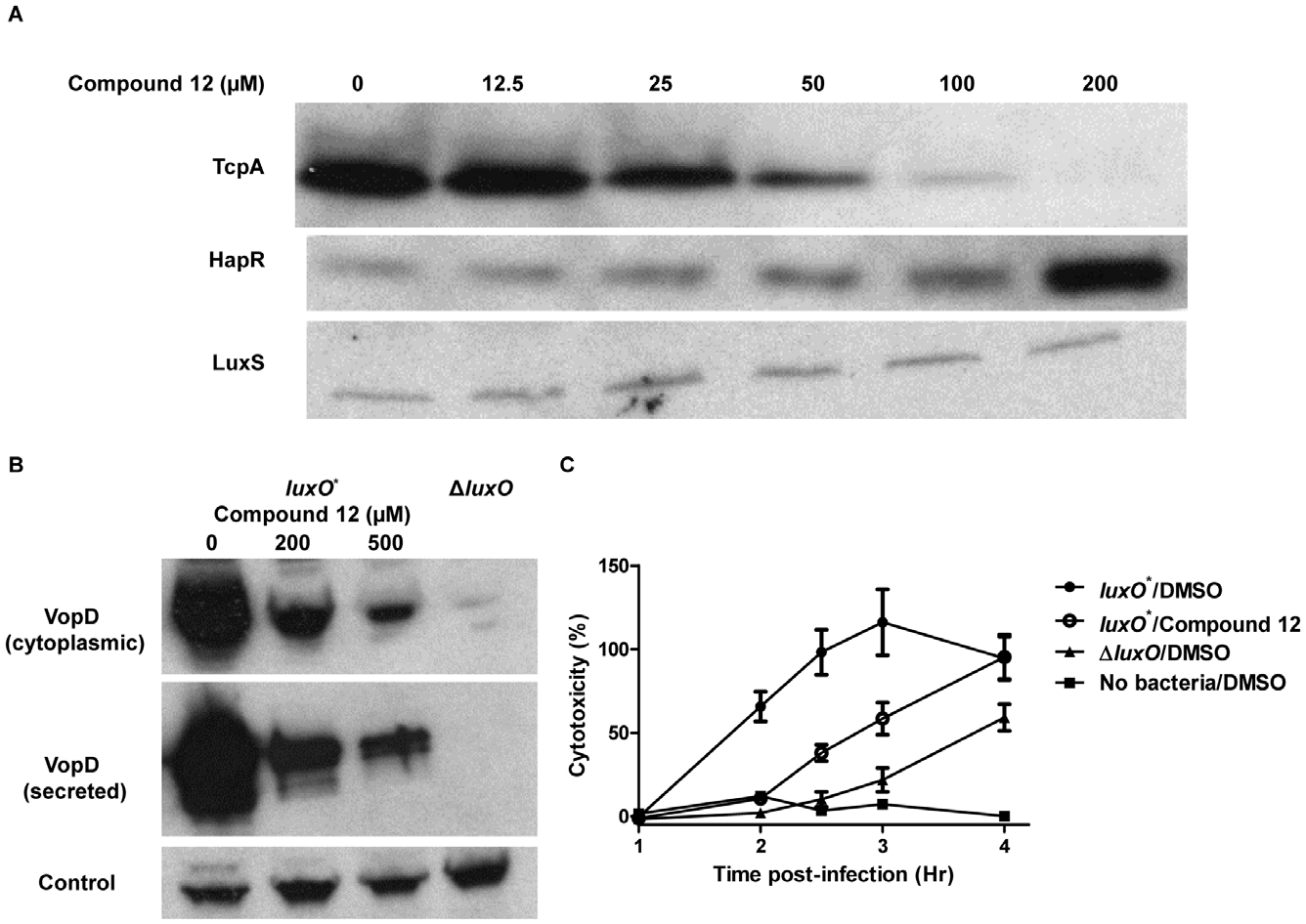
Control of virulence factor production by LuxO inhibitors. (A) Western blot analysis of TcpA (Top), HapR (middle), and LuxS (bottom, loading control) in a *V. cholerae luxO* ^D47E^ mutant in the presence of 0, 12.5, 25, 50, 100, and 200 µM compound 12. (B) Western blot analysis of the cytoplasmic and secreted VopD in the *V. parahaemolyticus* constitutively active luxO^*^ strain (LM4476) in the presence of 0, 200, and 500 µM compound 12. An isogenic *V. parahaemolyticus* Δ*luxO* mutant (LM9968) is included as the control. (C) Cytotoxicity of *V. parahaemolyticus* LM4476 (*luxO* ^*^) on cultured HeLa cells in the absence and presence of 500 µM compound 12. Cytotoxicity was measured by lactate dehydrogenase (LDH) release from HeLa cells. 100% cytotoxicity denotes LDH activity released upon treatment with 0.45% (v/v) Triton-X100. The *V. parahaemolyticus* Δ*luxO* mutant LM9968 and the no-bacteria control are included for comparison. Error bars represent standard errors of the mean for three independent trials.

In pathogenic vibrios, HapR and its homologs (e.g., *V. parahaemolyticus* OpaR and *V. vulnificus* SmcR) function as repressors of virulence factor production at HCD [32]–[34]. For example, in *V. cholerae*, the genes encoding the key *V. cholerae* virulence factors, the CTX toxin and the Toxin Co-regulated Pilus (TCP), are targets of HapR repression at HCD [17], [27], [30]. *V. parahaemolyticus* uses Type Three Secretion Systems (TTSS) for pathogenesis, and at HCD, OpaR represses the expression of one of the TTSS operons (TTSS-1) [32], [47]. Thus, *luxO* mutants that constitutively produce HapR (*V. cholerae*) or OpaR (*V. parahaemolyticus*) are attenuated in virulence [22], [30], [32]. The previous section shows that our LuxO inhibitors are active in multiple vibrios. To test whether the inhibitors can disrupt the QS-controlled virulence outputs of pathogenic vibrios, we assayed their effects on TcpA production in *V. cholerae* and production and secretion of VopD, a TTSS-1 effector protein, in *V. parahaemolyticus*. Western blot analysis showed that, in a *V. cholerae luxO* ^D47E^ strain, HapR and TcpA levels increased and decreased, respectively, in the presence of compound 12 (Figure 8A). Likewise, exposing the V. parahaemolyticus luxO^*^ mutant to compound 12 resulted in decreased production and secretion of VopD (Figure 8B).

To begin to explore whether repression of these *in vitro* virulence phenotype translates to repression of the *in vivo* phenotype, we exploited an established *V. parahaemolyticus* cytotoxicity assay [48] to investigate whether pathogenicity could be inhibited by treatment with the LuxO inhibitors. We infected cultured HeLa cells with the untreated or compound 12-treated V. parahaemolyticus luxO^*^ mutant and assayed HeLa cell lysis by measuring lactate dehydrogenase released from the host cytoplasm. Consistent with a previous report [32], the V. parahaemolyticus luxO* mutant is more cytotoxic to HeLa cells than the isogenic Δ*luxO* mutant (Figure 8C). At 2 to 3 hours post-infection, HeLa cell lysis was significantly lower in samples infected with the luxO^*^ mutant treated with compound 12 than in samples infected with the luxO^*^ mutant that had not been treated (average cytotoxicity is ∼30% and ∼100% for treated and untreated, respectively, p<0.01). At that time point, the cytotoxic capability of the Compound 12-treated luxO^*^ mutant is slightly higher than that of the isogenic Δ*luxO* mutant (Figure 8C). At 4-hour post-infection, the compound 12-treated luxO^*^ mutant was equally toxic (∼100%) as the untreated the luxO^*^ mutant, while the Δ*luxO* mutant caused only ∼60% HeLa cells lysis. This residual cytotoxicity is consistent with earlier results showing that the Δ*luxO* mutant is not completely impaired for cytotoxicity [32]. Thus, the level of *in vitro* inhibition of TTSS-1 (Figure 8B) is a good indicator of the *ex vivo* inhibition of cytotoxicity (Figure 8C). The increase in cytotoxicity in Compound 12-treated *V. parahaemolyticus* that occurred at late time points could be due to incomplete inhibition of LuxO, uptake, or degradation of the compound by the HeLa cells. Nonetheless, the progression of *V. parahaemolyticus* killing of mammalian cells is impaired by compound 12, consistent with the notion that virulence factor production can be controlled by small molecule inhibitors of LuxO.

## Discussion

As part of a continuing effort to identify molecules that modulate QS in bacteria, we have identified two classes of molecules that activate QS in *V. cholerae*. These newly identified molecules serve two important purposes. First, they can be used as novel chemical probes to study QS signal transduction mechanisms. Second, from a practical standpoint, because QS represses virulence factor production in many pathogenic *Vibrio* species, molecules that activate QS, which decreases virulence, have the potential to be developed into anti-virulence agents to combat infectious diseases caused by pathogenic vibrios.

The first class of molecules identified here acts on the *V. cholerae* CqsS receptor. These molecules, surprisingly, do not resemble the native CAI-1 family of ligands (Figure 2A). Previous studies revealed that CqsS receptors from different vibrios possess distinct ligand detection specificities. The *V. cholerae* receptor is promiscuous in detecting a range of CAI-1-type molecules, while the *V. harveyi* receptor is relatively stringent [39]. Interestingly, none of the Class 1 molecules identified here activates QS in *V. harveyi*, lending support to the idea that CqsS receptors, although sharing extensive homology, possess different overall stringencies for ligands. We altered a single specificity-determining residue in the *V. cholerae* CqsS receptor (Cys 170) to the corresponding amino acid (Phe) in the *V. harveyi* receptor. This alteration is sufficient to increase stringency in detection of CAI-1 type molecules [39], [49], however, it did not abolish detection of the Class 1 molecules (Figure S4). Identification of CqsS receptor mutants with altered selectivity to the Class 1 molecules will provide additional insight into the molecular basis of ligand-CqsS interactions.

The second class of molecules identified, and the focus of this work, act on LuxO, the central QS regulator that controls transcription of the four Qrr sRNA genes. LuxO, which is a member of the NtrC family of two-component response regulators, possesses an N-terminal regulatory receiver domain, a central ATPase domain (AAA+ type), and a C-terminal DNA-binding domain. Three inhibitors have previously been identified that target non-NtrC type response regulators, AlgR1 of *Pseudomonas aeruginosa* [50], WalR in low-GC Gram-positive bacteria [51], and DevR in *Mycobacterium tuberculosis* [52]. The molecules function by perturbing phosphorylation (AlgR1 and WalR) and DNA binding (DevR). Our LuxO inhibitors, by contrast, function by an uncompetitive mechanism, presumably by binding to the pre-formed LuxO-ATP complex to prevent ATP hydrolysis. Thus, multiple families of response regulator can be selectively inhibited using small molecules. Furthermore, all three known response regulator activities; phosphorylation, DNA binding, and ATPase, are potential targets for inhibition. Analyses of LuxO inhibitor-resistant mutants suggest that our inhibitors bind to a region close to the predicted Walker B motif. Additional support for this idea comes from studies of other NtrC-type proteins, which show that mutations that affect ATP hydrolysis but do not interfere with ATP binding also map to the Walker B motif and to amino acid residues preceding the conserved GAFTGA domain [43], [53], [54]. Indeed, one of the LuxO inhibitor-resistant mutations identified here (L242F) lies immediately upstream of the predicted Walker B motif, while both the I211F and L215F mutations map to the helix containing the GAFTGA domain. In addition, the residue identified in the final inhibitor-resistant mutant, V294L, is predicted to sit facing the putative catalytic arginine (R306). The GAFTGA domain is important for interaction with the σ^54^-RNAP holoenzyme [55]. Thus, it was possible that the mutations we isolated in this region (I211F and L215F) suppress inhibition by compounds 11 and 12 by stabilizing the LuxO-σ^54^-RNAP interaction without affecting inhibitor binding. If this were the case, the ATPase activity of the purified LuxO D47E/I211F and D47E/L215F variants would be inhibited by these compounds. However, we purified LuxO D47E/I211F protein and found that the ATPase activity is not inhibited (Figure S5). This result is consistent with the idea that these mutations abolish inhibitor binding and, in so doing, prevent ATP hydrolysis.

High sequence conservation in the ATPase domain exists between different NtrC-type response regulator family members. Thus, we were interested to test whether the LuxO inhibitors could inhibit other NtrC-type response regulators. Compounds 11 and 12 only modestly inhibit (∼10%) the ATPase activity of purified *E. coli* NtrC at 250 µM (a concentration at which >80% of the LuxO ATPase activity is inhibited, Figure S6). This finding is surprising because the key residues (I211, L215, L242, and V294) that, when mutated, confer resistance to the inhibitors in LuxO are all present in *E. coli* NtrC. Thus, NtrC must possess additional structural features that render it resistant to inhibition. Structural comparisons between these two related RRs, coupled with identification of inhibitor-sensitive NtrC mutants, should allow us to understand the basis of the differences in inhibitor sensitivity.

Two-component signaling (TCS) proteins are widely distributed in bacteria. In addition to their global importance in microbial physiology, the absence of TCSs in mammalian cells makes them attractive drug targets in pathogenic bacteria [56], [57]. Even though significant effort has been devoted to identifying novel TCS inhibitors, to date, none has been developed into a new class of anti-infective. Problems such as undesirable properties associated with lead molecules have been encountered [56], [57]. In particular, inhibitors that generally target the conserved hydrophobic kinase domains of TCS histidine kinases suffer from drawbacks such as low cell permeability, poor selectivity, and unfavorable non-specific off-target effects (e.g. membrane damaging) [58]–[60]. By contrast, approaches to target the sensory domains of histidine kinases have yielded a handful of promising TCS inhibitors. For instance, LED209, an antagonist of the QseC histidine kinase, which regulates motility and pathogenicity in enterohaemorrhagic *E. coli*, reduces virulence in several pathogens both *in vitro* and *in vivo* [61]. In addition, in *Staphylococcus aureus*, inhibitory Agr peptide analogs antagonize the AgrC histidine kinase receptors and block abscess formation in an experimental murine model [62].

Targeting response regulators as a broad-spectrum anti-infective strategy has been considered challenging because response regulator functions, such as phosphorylation and DNA binding, are thought to be specific. In spite of this, a handful of molecules that inhibit particular response regulator functions have been reported [50]–[52]. For example, as mentioned, Walrycins, molecules that inhibit the phosphorylation of the essential WalR response regulator, are active in suppressing growth in multiple Gram-positive bacteria [51]. In the context of our work, the ATPase domain is highly conserved between all members of the NtrC response regulator family. Therefore, molecules that specifically target the ATPase domain of a single response regulator in this family (e.g., LuxO) could potentially be developed into general inhibitors of NtrC-family of proteins. Because NtrC-type proteins control virulence, nitrogen metabolism, motility, and other vital processes in bacteria [37], targeting the ATPase domain offers an additional route for anti-TCS drug development.

The LuxO inhibitors identified here possess certain favorable drug-like characteristics: potent inhibition, water-solubility, good stability, and cell-permeability. The molecules also display low host-cell cytotoxicity (undetectable cytotoxicity at 500 µM). These broadly-active LuxO inhibitors are not broad-spectrum NtrC-type inhibitors. Microarray analyses reveal that fewer than 40% of genes affected by the inhibitors are non-LuxO targets (data not shown). Nonetheless, our LuxO inhibitors could be used as preliminary scaffolds for building a general NtrC-type RRs inhibitor. Future improvements to these molecules will be focused on the structure-activity relationships of the thio-azauracil core, combined with simultaneously screening for molecules that inhibit LuxO and other NtrC type response regulators.

Although NtrC is not affected by the inhibitors discovered here, multiple LuxO response regulators from different *Vibrio* species are targeted by our inhibitors. *Vibrio* species detect a wide array of autoinducers (HSLs, CAI-1, and AI-2), thus, molecules that interrupt QS in *Vibrio* species by targeting the cognate receptors/synthases are likely to be autoinducer-specific and will have a limited spectrum. By contrast, because LuxO is nearly identical in all *Vibrio* species, our inhibitors can broadly activate vibrio QS irrespective of what type of autoinducer is detected. More importantly, we showed here that treatment of *V. cholerae* and *V. parahaemolyticus* with the LuxO inhibitors reduces virulence factor production and impedes cytotoxicity. Thus, our LuxO inhibitors, upon refinement, can at a minimum be used broadly to control virulence factor production in a variety of *Vibrio* species that use QS to repress pathogenesis.

The central ATPase module of the NtrC-type RR is classified as AAA+ type [63]. This module is present in multiple domains of life. For example, AAA+ ATPases are important in functions including protein unfolding and degradation (ClpXP, FtsH, and p97), organelle function and maintenance (PEX1 and VPS4), replication and recombination (RuvBL1 and helicases), and intracellular transport (Dyneins). Some eukaryotic AAA+ ATPases have been proposed to be drug targets [64]. Therefore, it will be particularly fascinating to investigate whether the thio-azauracil core discovered here can be developed into an inhibitor of AAA+ ATPases across different domains.

Antagonizing QS in bacteria represents a promising new approach that is an alternative to traditional antibiotics [8], [12], [14], [15], [61], [65]. Likewise, using pro-QS agents to treat acute infections, in which bacteria use QS to repress virulence, should be further explored. Using the native CAI-1 ligand, we previously showed that *V. cholerae* virulence factor production is repressed *in vitro* [17]. In the same vein, we show here that our synthetic pro-QS molecules reduce virulence by inhibiting LuxO. March *et al* reported that pretreatment with commensal *E. coli* over-producing the *V. cholerae* autoinducer CAI-1 increases the survival rate of mice following *V. cholerae* infection [66], which further supports the idea of QS potentiators as drugs. Use of CAI-1, LuxO inhibitors, or other QS-activating molecules as prophylactics could conceivably prevent *V. cholerae* or other pathogenic vibrios from initiating the LCD virulence gene expression program that is required for colonization. In this scenario, inhibiting the launch of virulence factors would provide sufficient time for the host immune system to eliminate the pathogen. In contrast to traditional antibiotics that target essential bacterial processes, growth is not affected by interfering with QS, so development of resistance could potentially be minimized [8], [14].

## Materials and Methods

### Bacterial strains and culture conditions

All *V. cholerae* strains are derivatives of wild type C6706str [67]. All *V. harveyi* strains are derivatives of wild type *V. harveyi* BB120 [68]. *V. parahaemolyticus* strains were generously provided by Dr. Linda McCarter. *Escherichia coli* S17-1 p*ir*, DH5α, and Top10 were used for cloning. The relevant genotypes of all plasmids and strains are provided in Supporting Table S1. Unless specified, *E. coli* and *V. cholerae* were grown in LB medium at 37°C and 30°C with shaking, respectively. *V. harveyi and V. parahaemolyticus* were grown in LM medium at 30°C with shaking. Colony opacity of *V. parahaemolyticus* was monitored on LM with 2% agar. Unless specified, antibiotic concentrations are as follows: ampicillin, gentamicin, and kanamycin, 100 mg/L; chloramphenicol and tetracycline, 10 mg/L; streptomycin, 5 g/L; polymyxin B, 50 U/L.

### Screening for *V. cholerae* QS-activating molecules

The 90,000 molecule library was supplied by the High-Throughput Screening Resource Center of the Rockefeller University. The *V. cholerae* strains BH1578 (Δ*cqsA* Δ*luxS* pBB1) and BH1651 (*luxO* ^D47E^ pBB1) were grown overnight in LB medium with tetracycline and diluted 25-fold. The diluted cultures were dispensed into 384-well microtiter plates containing screening molecules that were previously added to each well. The final concentration of each compound was ∼20 µM. Light production was measured on an Envison Multilabel Reader after 6-hour incubation at 30°C without shaking. Compounds that induced light production >100-fold were reordered from suppliers and tested.

### Bioluminescence assays for *V. cholerae* and *V. harveyi*

Overnight cultures of reporter strains were grown in LM medium (for *V. harveyi*) or LB with tetracycline (for *V. cholerae* carrying pBB1) and diluted 20-fold with sterile medium. Bioluminescence and OD_600_ were measured in an Envison Multilabel Reader following 4-hour incubation at 30°C with shaking. Synthetic molecules were dissolved in DMSO and supplied at varying concentrations to the reporter strains. DMSO was used as the negative control.

### Protein purification

The open reading frame encoding *V. cholerae* LuxO D47E was amplified by PCR and cloned into plasmid pET28B that had been previously digested with NdeI and BamHI. The resulting plasmid was transformed into *E. coli* BL21 Gold (DE3) resulting in strain WN133. Strain WN133 was grown in LB with kanamycin at 30°C with shaking until the OD_600_ of the culture reached ∼1.0. IPTG was added at a final concentration of 200 µM, and the culture was incubated for an additional 4 hours at 30°C with shaking. Cells were harvested by centrifugation, suspended in lysis buffer (20 mM Sodium phosphate buffer pH 7.4, 0.5 M NaCl, 10% glycerol, and 5 mM imidazole), and lysed using a Cell Cracker. Soluble materials were loaded onto a HiTrap chelating column charged with nickel, the column was washed extensively with lysis buffer, and His_6_-tagged *V. cholerae* LuxO D47E enzyme was eluted using a linear gradient of increasing concentration of imidazole dissolved in lysis buffer. Fractions containing LuxO D47E were pooled and concentrated with an Amicon Untra-15 filter. Protein was snap-frozen in liquid nitrogen and stored at −80°C. Protein concentrations were determined by UV absorbance at 280 nm. *E. coli* NtrC and other LuxO D47E variants were purified using the same method.

### ATPase assays

A modified coupled-enzyme assay was used to measure the rate of ATP hydrolysis by LuxO D47E [42]. Briefly, ADP released from ATP by LuxO D47E is reacted with phosphoenolpyruvate (PEP) to form pyruvate using pyruvate kinase (PK). Pyruvate is reacted with NADH to form NAD and lactate using lactate dehydrogenase (LDH). The rate of NAD production is followed at 340 nm using a spectrophotometer. ATP hydrolysis rates were inferred from the absorbance change observed (ε_NADH,340_−ε_NAD,340_ = 6220 M^−1^ cm^−1^ for NADH) [42]. The rates of ATP hydrolysis by LuxO D47E were measured in reactions containing 100 mM Sodium phosphate buffer pH 7.4, 5 mM MgCl_2_, 0.2 mM NADH, 1 mM PEP, 5–20 units of PK/LDH mix (Sigma), and 10 µM LuxO D47E. ATP and inhibitors were added to the reactions at indicated concentrations. The rate of ATP hydrolysis was monitored for 5 minutes. Data were fitted using Graphpad Prism to obtain the kinetic parameters. Percent ATPase inhibition was calculated using the following formula:
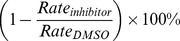



### DNA binding assays

Electrophoretic mobility shift assays to study LuxO and Qrr promoter DNA interactions were performed as described in [69]. Fluorescence anisotropy assays using LuxO D47E were modified from [70].

### Screening for LuxO mutants resistant to inhibitors

The *luxO^D47E^* allele was removed from plasmids harbored in WN133 with the enzymes XbaI and BamHI and ligated into pEVS143 [71] that had been previously digested with AvrII and BamHI. The *luxO^D47E^* reading frame of the resulting plasmid (WN2029) was randomly mutated using the GeneMorph II Random Mutagenesis Kit. The resulting mutagenized *luxO^D47E^* plasmid library was introduced into a *V. cholerae* Δ*luxO* strain by conjugation. Individual colonies from this *V. cholerae luxO^D47E^* mutant pool were arrayed into 96-well plates containing LB medium with 100 µM compound 12. The *V. cholerae* Δ*luxO* strain harboring non-mutated *luxO* ^D47E^ was grown in the absence of compound 12 to provide the reference for background light production. Following overnight static incubation at 30°C, clones that produced light comparable to the background were selected and re-tested in the presence and absence of compounds 11 and 12. DNA sequencing was used to determine the alterations in *luxO* ^D47E^ for inhibitor-resistant mutants. Site-directed mutageneses were performed with the QuikChange II XL Site-Directed Mutagenesis Kit to uncouple multiple mutations.

### Western blot analysis

Overnight cultures of the *V. cholerae luxO* ^D47E^ strain were diluted 1000-fold in AKI medium containing the indicated concentrations of compound 12. The cultures were statically incubated at 37°C for 4 hours and subsequently shaken for 4 more hours at 37°C. Cells were collected by centrifugation, TcpA from different samples was analyzed by Western blot as previously described [17]. Overnight cultures of the V. parahaemolyticus luxO* strain (LM4476) were washed and diluted 50-fold in LM medium with 10 mM MgCl_2_ and 10 mM sodium oxalate in the presence of the indicated concentrations of compound 12. The cultures were grown for 4 hours with shaking at 37°C. Viable cell count showed that all cultures contained ∼1×10^9^ CFU/mL after incubation. Cells were collected by centrifugation, and the secreted and cytoplasmic VopD from different samples were analyzed by Western blot as previously described [47].

### Cytotoxicity assays

Cytotoxicity assays were modified as previously described [48]. HeLa cells (2×10^4^ cells/well) were cultured for 48 hours at 37°C and 5% CO_2_ in a 96-well plate containing DMEM with 10% fetal bovine serum prior to infection. *V. parahaemolyticus* strains were grown as described above for VopD analysis and used in the infection assays. Immediately prior to *V. parahaemolyticus* infection, DMSO or compound 12 (500 µM) was added to the HeLa. Serially diluted bacteria were added to HeLa cells at multiplicity of infection of 10. Lactate dehydrogenase release from HeLa cells was assayed between 1–4 hours after infection using the CytoTox 96 nonradioactive cytotoxicity kit (Promega).

### Chemical synthesis and analytical methods

All chemical syntheses and analytical methods are provided in the Supporting Text S1.

## Supporting Information

Figure S1 **The effect of QS modulators on growth.** To test if QS modulators affect growth, *V. cholerae* strain BH1578 was incubated with 100 µM of compounds 1 to 12. Optical density at 600 nm was monitored thereafter for a total of 4 hours of incubation at 30°C. No significant difference was observed between the DMSO control and the treatments. Error bars represent standard errors of the means from three independent samples.
(TIF)

Figure S2 **Responses to Class 1 compounds by** ***Vibrio cholerae*** **strains lacking each QS receptor.** To determine which QS receptor each Class 1 compound acts on, we tested the eight Class 1 compounds against *V. cholerae* mutants lacking only the CqsS receptor (black bars) or only the LuxPQ receptor (white bars). All eight Class 1 compounds induced light production in the Δ*luxPQ* strain but not the Δ*cqsS* strain. Normalized light production (RLU) was measured in *V. cholerae* strains lacking either the CqsS or the LuxPQ QS receptor in the presence of 50 µM of the Class 1 compounds. Error bars represent standard errors of the means from three independent samples.
(TIF)

Figure S3 **The effect of** ***luxO*** **mutations on inhibitor resistance in the wild type LuxO protein.** The *luxO m*utations I211F, L215F, L242F, and V294L that confer inhibitor resistance were individually introduced into the plasmid carrying wild type *luxO*. The resulting plasmids were mobilized into a *V.cholerae* Δ*cqsA* Δ*luxS* Δ*luxO* mutant carrying the heterologous *V. harveyi lux* operon. In strain expressing wild type LuxO, the inhibitors (100 µM compounds 11 and 12) were capable of inhibiting LuxO, thus, light production was induced >5000-fold (grey and black bars). By contrast, light production was only induced at ≤300-fold in the LuxO mutants I211F, L215F, L242F, and V294L, suggesting these *luxO* mutations confer resistance to the inhibitors in the context of the wild type protein.
(TIF)

Figure S4 **Responses to Class 1 compounds by** ***Vibrio cholerae*** **CqsS mutants with altered receptor specificities.** Previous studies showed that the CqsS C170Y mutation causes increased specificity for a ligand with a C10 tail and an overall reduction in sensitivity to CAI-1. Normalized light production (RLU) was measured in *V. cholerae* strains carrying wild type CqsS (WN1103) or the CqsS^C170Y^ receptor (WN1992) in the presence of 50 µM of the Class 1 compounds. Error bars represent standard errors of the means from three independent samples. The results show that the C170Y mutation does not abolish detection of some of the Class 1 compounds (e.g., cpd1, cpd 3, and cpd 11).
(TIF)

Figure S5 **ATPase activity of LuxO D47E and LuxO D47E/I211F in the presence of the LuxO inhibitors.** Mutations I211F and L215F map in close proximity to the LuxO GAFTGA domain, which is presumed to be required for interaction with RNA polymerase (RNAP). Therefore, it was possible that mutations causing insensitivity to the Class 2 compounds could suppress inhibition by stabilizing the LuxO-σ^54^-RNAP interaction without affecting inhibitor binding. If this were the case, the ATPase activity of LuxO D47E/I211F and D47E/L215F variants would remain inhibited by these compounds. The experiment below shows that while the ATPase activity of LuxO D47E is inhibited by the compounds (open and closed circles), the ATPase activity of the purified LuxO D47E/I211F protein is not affected (open and closed squares). ATP hydrolysis was measured using a coupled-enzyme assay that monitors changes in absorbance at 340 nm. 100 µM of Compound 12 and 2.5 mM ATP were used in the assay.
(TIF)

Figure S6 **The effect of LuxO inhibitors on** ***E. coli*** **NtrC.** High sequence conservation in the ATPase domain exists between different NtrC-type response regulators. To test whether the Class 2 LuxO inhibitors also inhibit other NtrC-type response regulators, we examined *E. coli* NtrC. While >80% of the LuxO ATPase activity is inhibited (open and closed circles) by 250 µM of compound 11, the inhibitor only modestly inhibits (∼10%) the ATPase activity of purified *E. coli* NtrC D54E (open and closed squares).
(TIF)

Table S1 **Bacterial strains used in this study.**
(DOCX)

Text S1 **Chemical Synthesis and Analytical Methods.**
(DOCX)
