# Supplementary material for: Broad Spectrum Pro-Quorum-Sensing Molecules as Inhibitors of Virulence in Vibrios
Source: PLoS Pathog. 2012 Jun 28;8(6):e1002767. doi: 10.1371/journal.ppat.1002767 (PMC3386246; doi:10.1371/journal.ppat.1002767)
Supplement: Table S1 — Bacterial strains used in this study. (DOCX) [file ppat.1002767.s007.docx]

**Table S1 Strain Table**

| **Strain^a^** | **Genotype** | **Plasmid** | **Reference** |
| --- | --- | --- | --- |
| ***Vibrio cholerae*** | | | |
| BH1578 | Δ*cqsA* Δ*luxS* | pBB1 (*luxCDABE* from *V. harveyi*) | Hammer |
| BH1651 | *luxO*^D47E^ | pBB1 | Hammer |
| BH1644 | *luxO*^D47E^ |  | Hammer |
| SLS349 | Δ*luxO* |  | Svenningsen |
| WN1103 | Δ*cqsA* Δ*luxPQ* | pBB1 | This study |
| WN1992 | Δ*cqsA cqsS*^C170Y^ Δ*luxPQ* | pBB1 | This study |
| DH231 | Δ*luxS* Δ*cqsS* | pBB1 | This study |
| SLS353 | *luxO*^D47E^ | pSLS4 (*qrr*4-*gfp*) | Svenningsen |
| SLS373 | Δ*luxO* | pSLS4 (*qrr*4-*gfp*) | Svenningsen |
| WN2442 | Δ*luxO* | pEVS143-LuxO D47E and pBB1 | This study |
| WN2525 | Δ*luxO* | pEVS143-LuxO D47E/I211F and pBB1 | This study |
| WN2527 | Δ*luxO* | pEVS143- LuxO D47E/L215F and pBB1 | This study |
| WN2531 | Δ*luxO* | pEVS143- LuxO D47E/V294L and pBB1 | This study |
| WN2579 | Δ*luxO* | pEVS143- LuxO D47E/L242F and pBB1 | This study |
| WN2632 | Δ*cqsA* Δ*luxS* Δ*luxO* | pEVS143-LuxO and pBB1 | This study |
| WN2634 | Δ*cqsA* Δ*luxS* Δ*luxO* | pEVS143-LuxO I211F and pBB1 | This study |
| WN2636 | Δ*cqsA* Δ*luxS* Δ*luxO* | pEVS143- LuxO L215F and pBB1 | This study |
| WN2638 | Δ*cqsA* Δ*luxS* Δ*luxO* | pEVS143- LuxO V294L and pBB1 | This study |
| WN2640 | Δ*cqsA* Δ*luxS* Δ*luxO* | pEVS143- LuxO L242F and pBB1 | This study |
| ***Vibrio harveyi*** | | | |
| KM83 | *luxO*^D47E^ |  | Tu |
| WN1492 | Δ*cqsA* Δ*cqsS* Δ*luxN* Δ*luxPQ* | pLAFR-CqsS | Ng |
| WN1834 | Δ*cqsA* Δ*cqsS* Δ*luxN* Δ*luxPQ* | pLAFR-CqsS F175C | Ng |
| ***Vibrio parahaemolyticus*** | | | |
| LM4476 | *luxO*^*^ |  | Gode-Potratz |
| LM9688 | Δ*luxO* |  | Gode-Potratz |
| ***E. coli*** | | | |
| WN133 | BL21 (DE3) | pET28B-LuxO D47E | This study |
| WN2600 | BL21 (DE3) | pET28B-LuxO D47E/I211F | This study |
| WN2181 | BL21 (DE3) | pET28B-NtrC D54E | This study |

**^a^** The alteration in the *luxO*^D47E^ allele is Asp61Glu, while other alterations (I211, L215, V294, and L242) in LuxO represent the exactly numbered residue. The *luxO*^D47E^ allele nomenclature is maintained for consistency in this study and to reflect the nomenclature that is conventionally used in the literature.

**References**

Gode-Potratz, C.J., and McCarter, L.L. (2011). Quorum sensing and silencing in *Vibrio parahaemolyticus*. J Bacteriol 193, 4224-4237.

Hammer, B.K., and Bassler, B.L. (2007). Regulatory small RNAs circumvent the conventional quorum sensing pathway in pandemic *Vibrio cholerae*. Proc Natl Acad Sci U S A 104, 11145-11149.

Ng, W.L., Wei, Y., Perez, L.J., Cong, J., Long, T., Koch, M., Semmelhack, M.F., Wingreen, N.S., and Bassler, B.L. (2010). Probing bacterial transmembrane histidine kinase receptor-ligand interactions with natural and synthetic molecules. Proc Natl Acad Sci U S A 107, 5575-5580.

Svenningsen, S.L., Waters, C.M., and Bassler, B.L. (2008). A negative feedback loop involving small RNAs accelerates *Vibrio cholerae*'s transition out of quorum-sensing mode. Genes Dev 22, 226-238.

Tu, K.C., and Bassler, B.L. (2007). Multiple small RNAs act additively to integrate sensory information and control quorum sensing in *Vibrio harveyi*. Genes Dev 21, 221-233.
