# Supplementary material for: Broad Spectrum Pro-Quorum-Sensing Molecules as Inhibitors of Virulence in Vibrios
Source: PLoS Pathog. 2012 Jun 28;8(6):e1002767. doi: 10.1371/journal.ppat.1002767 (PMC3386246; doi:10.1371/journal.ppat.1002767)
Supplement: Text S1 — Chemical Synthesis and Analytical Methods. (DOCX) [file ppat.1002767.s008.docx]

**Support Text S1: Chemical Synthesis and Analytical Methods**

**Analytical methods.** NMR spectra were recorded using a Bruker Avance II spectrometer (500 MHz for ^1^H; 125 MHz for ^13^C) equipped with either a ^1^H-optimized TCI (H/C/N) cryoprobe or a ^13^C-optimized dual C/H cryoprobe. Chemical shifts are reported in parts per million (ppm) and were calibrated according to residual solvent. High-resolution mass spectral analysis was performed using an Agilent 1200-series electrospray ionization – time-of-flight (ESI-TOF) mass spectrometer in the positive ESI mode.

**Chemical Reactions.** Unless otherwise noted, all reactions were performed in flame-dried glassware under an atmosphere of nitrogen. All chemicals purchased from commercial vendors were used without further purification. Anhydrous Sure/Seal^TM^ solvents were purchased from commercial vendors.

**Purification.** Flash chromatography was performed using C18 Sep-Pak Cartridges from Waters Corporation. Analytical thin-layer chromatography was carried out using Silica G TLC plates, 200 μm with UV_254_ fluorescent indicator (SORBENT Technologies), and visualization was performed by staining (anisaldehyde, ceric ammonium molybdate, or ninhydrin) and/or by absorbance of UV light.

**Compound Synthesis.**

**
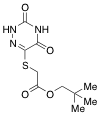
**

**General Procedure: Neopentyl 2-((3,5-dioxo-2,3,4,5-tetrahydro-1,2,4-triazin-6-yl)thio)acetate, Compound 12.** To 2,2-dimethylpropan-1-ol (260 μL, 2.4 mmol) and Et_3_N (335 μL, 2.4 mmol) in CH_2_Cl_2_ (4.8 mL) at 0 ^o^C was added chloroacetyl chloride (190 μL, 2.4 mmol). The mixture was allowed to stir with warming to ambient temperature over 4 h. and was quenched with H_2_O (20 mL), extracted with CH_2_Cl_2_ (2 x 20 mL), washed with 1N HCl (20 mL), sat. NaHCO_3_ (20 mL), brine (20 mL), dried over Na_2_SO_4_, and concentrated *in vacuo*. The resulting colorless oil was used without further purification. To the crude neopentyl 2-chloroacetate (395 mg, 2.4 mmol) in EtOH (2.4 mL) at room temperature was added sodium 3,5-dioxo-2,3,4,5-tetrahydro-1,2,4-triazine-6-thiolate (freshly prepared from 6-mercapto-1,2,4-triazine-3,5(2*H*,4*H*)-dione^1^ (348 mg, 2.4 mmol) and NaOH (115 mg, 2.9 mmol) in H_2_O (4.8 mL) at room temperature for 1 h.). The resulting mixture was allowed to stir at 40 ^o^C for 14 h. and was diluted with H_2_O (5 mL) before loading directly onto a 10g C18 Sep-Pak Cartridge and elution with H_2_O (25 mL) followed by 1:1 H_2_O:MeOH (25 mL) to yield neopentyl 2-((3,5-dioxo-2,3,4,5-tetrahydro-1,2,4-triazin-6-yl)thio)acetate as a white solid (127 mg, 19% yield over two steps). ^1^H-NMR (500 MHz, *d*4-MeOH) δ 3.81 (s, 2H), 3.78 (s, 2H), 0.92 (s, 9H). ^13^C-NMR (125MHz, CDCl_3_) δ 171.3, 167.1, 161.6, 145.4, 75.9, 32.4, 32.1, 26.8. HRMS (ESI-TOF) calculated for C_10_H_16_N_3_O_4_S, 274.0862; observed 274.0860 [M+H]^+^

**
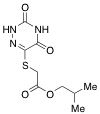
**

**Isobutyl 2-((3,5-dioxo-2,3,4,5-tetrahydro-1,2,4-triazin-6-yl)thio)acetate, Compound 11.** Prepared following the general procedure from 2-methylpropan-1-ol and 6-mercapto-1,2,4-triazine-3,5(2*H*,4*H*)-dione (21 mg, 12% yield over two steps). ^1^H-NMR (500 MHz, *d*4-MeOH) δ 3.90 (d, *J*= 6.6 Hz, 2H), 3.77 (s, 2H), 1.92 (septet, *J*= 6.7 Hz, 1H), 0.92 (d, *J*= 6.7 Hz, 6H). ^13^C-NMR (125MHz, CDCl_3_) δ 171.2, 166.0, 160.4, 145.6, 72.8, 32.2, 29.1, 19.5. HRMS (ESI-TOF) calculated for C_9_H_14_N_3_O_4_S, 260.0705; observed 260.0701 [M+H]^+^

**
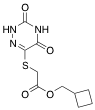
**

**Cyclobutylmethyl 2-((3,5-dioxo-2,3,4,5-tetrahydro-1,2,4-triazin-6-yl)thio)acetate, compound 13.** Prepared following the general procedure from cyclobutylmethanol and 6-mercapto-1,2,4-triazine-3,5(2*H*,4*H*)-dione (16 mg, 9% yield over two steps). ^1^H-NMR (500 MHz, *d*4-MeOH) δ 4.08 (d, *J*= 6.5 Hz, 2H), 3.31 (s, 2H), 2.67-2.58 (m, 1H), 2.08-1.98 (m, 2H), 1.96-1.71 (m, 4H). ^13^C-NMR (125MHz, CDCl_3_) δ 171.4, 167.1, 161.5, 145.5, 70.3, 35.6, 32.2, 25.7, 19.3. HRMS (ESI-TOF) calculated for C_10_H_14_N_3_O_4_S, 272.0705; observed 272.0700 [M+H]^+^

**
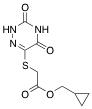
**

**Cyclopropylmethyl 2-((3,5-dioxo-2,3,4,5-tetrahydro-1,2,4-triazin-6-yl)thio)acetate, Compound 14.** Prepared following the general procedure from cyclopropylmethanol and 6-mercapto-1,2,4-triazine-3,5(2*H*,4*H*)-dione (12 mg, 7% yield over two steps). ^1^H-NMR (500 MHz, *d*4-MeOH) δ 3.80 (d, *J*= 7.2 Hz, 2H), 3.80 (s, 2H), 1.04-0.93 (m, 1H), 0.45-0.38 (m, 2H), 0.18-0.10 (m, 2H). ^13^C-NMR (125MHz, CDCl_3_) δ 171.4, 167.2, 161.6, 145.5, 71.5, 32.3, 10.8, 3.8. HRMS (ESI-TOF) calculated for C_9_H_12_N_3_O_4_S, 258.0549; observed 258.0553 [M+H]^+^

**
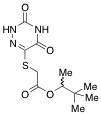
**

**3,3-Dimethylbutan-2-yl 2-((3,5-dioxo-2,3,4,5-tetrahydro-1,2,4-triazin-6-yl)thio)acetate, Compound 17.** Prepared following the general procedure from (±)-3,3-dimethylbutan-2-ol and 6-mercapto-1,2,4-triazine-3,5(2*H*,4*H*)-dione (45 mg, 20% yield over two steps). ^1^H-NMR (500 MHz, *d*4-MeOH) δ 4.66 (q, *J*= 6.4 Hz, 1H), 3.74 (s, 2H), 1.14 (d, *J*= 6.4 Hz, 3H), 0.9 (s, 9H). ^13^C-NMR (125MHz, CDCl_3_) δ 170.9, 166.9, 161.4, 145.5, 80.6, 35.3, 32.4, 26.2, 15.2. HRMS (ESI-TOF) calculated for C_11_H_18_N_3_O_4_S, 288.1018; observed 288.1011 [M+H]^+^

**
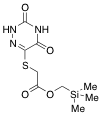
**

**(Trimethylsilyl)methyl 2-((3,5-dioxo-2,3,4,5-tetrahydro-1,2,4-triazin-6-yl)thio)acetate,** **Compound 16.** Prepared following the general procedure from (trimethylsilyl)methanol and 6-mercapto-1,2,4-triazine-3,5(2*H*,4*H*)-dione (45 mg, 23% yield over two steps). ^1^H-NMR (500 MHz, *d*4-MeOH) δ 3.83 (s, 2H), 3.76 (s, 2H), 0.06 (s, 9H). ^13^C-NMR (125MHz, CDCl_3_) δ 172.0, 167.0, 161.4, 145.5, 60.1, 32.0, -3.0. HRMS (ESI-TOF) calculated for C_9_H_16_N_3_O_4_SSi, 290.0631; observed 290.0627 [M+H]^+^

**
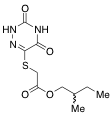
**

**3-Methylbutan-2-yl 2-((3,5-dioxo-2,3,4,5-tetrahydro-1,2,4-triazin-6-yl)thio)acetate,** **Compound 15.** Prepared following the general procedure from (±)-2-methyl-1-butanol and 6-mercapto-1,2,4-triazine-3,5(2*H*,4*H*)-dione (47 mg, 21% yield over two steps). ^1^H-NMR (500 MHz, *d*4-MeOH) δ 4.03-3.89 (m, 2H), 3.75 (s, 2H), 1.74-1.63 (m, 1H), 1.46-1.36 (m, 1H), 1.22-1.11 (m, 1H), 0.92-0.89 (m, 6H). ^13^C-NMR (125MHz, CDCl_3_) δ 171.4, 167.2, 161.6, 145.5, 71.2, 35.6, 32.2, 27.1, 16.8, 11.7. HRMS (ESI-TOF) calculated for C_10_H_16_N_3_O_4_S, 274.0862; observed 274.0858 [M+H]^+^

**
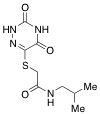
**

**2-((3,5-Dioxo-2,3,4,5-tetrahydro-1,2,4-triazin-6-yl) thio)-*N*-isobutylacetamide,** **Compound 18.** Prepared following the general procedure from 2-methylpropan-1-amine and 6-mercapto-1,2,4-triazine-3,5(2*H*,4*H*)-dione (27 mg, 13% yield over two steps). ^1^H-NMR (500 MHz, *d*4-MeOH) δ 4.56 (s, 2H), 3.64 (s, 2H), 3.05-2.92 (m, 2H), 1.77 (septet, *J*= 6.6 Hz, 1H), 0.90 (d, *J*= 6.6 Hz, 3H), 0.87 (d, *J*= 6.6 Hz, 3H). ^13^C-NMR (125MHz, CDCl_3_) δ 171.3, 170.8, 166.8, 159.6, 145.9, 55.1, 48.4, 48.1, 33.7, 29.8, 20.7, 20.6. HRMS (ESI-TOF) calculated for C_9_H_15_N_4_O_3_S, 259.0865; observed 259.0864 [M+H]^+^

^1^ Cristescu, C.; Andreescu, G. Ger. Offen. Patent DE 2022094, 1971.
